# Supplementary material for: Latent Class Analysis: Insights about design and analysis of schistosomiasis diagnostic studies
Source: PLoS Negl Trop Dis. 2021 Feb 4;15(2):e0009042. doi: 10.1371/journal.pntd.0009042 (PMC7888681; doi:10.1371/journal.pntd.0009042)
Supplement: S1 Appendix — Univariate and bivariate GF-Fits for the LC, LCRE and FM models for the Uganda data set. (PDF) [file pntd.0009042.s001.pdf]

S1 Appendix: Results (fit measures) for the Uganda data set

Table 1 gives the GF-Fit values for the pair POC-CCA and SEA-ELISA diagnostic tests and for the LC, LCRE and FM models for the Uganda data set.

Table 1: Contribution to GF-Fits: POC-CCA by SEA-ELISA, N=258

POC-CCA	SEA-ELISA	$N \times p_{ab}$	$N \times \hat{p}_{ab}$ LC	$N \times \hat{p}_{ab}$ LCRE	$N \times \hat{p}_{ab}$ FM
0	0	59	50.14	50.21	55.03
0	1	52	61.30	61.10	56.69
1	0	5	14.91	14.20	10.68
1	1	142	131.66	132.49	135.60
Total GF-Fit			10.37	9.54	4.00

Table 2 gives the univariate and bivariate GF-Fits for the LC, LCRE and FM models for all pairs of diagnostic tests. The univariate fits are in the diagonal and the bivariate fits are below the diagonal. Among the three models the LCRE provides the smallest bivariate GF-Fits but all models show adequate fit. Each value that appears in the table is smaller than 16.

Table 2: Univariate and Bivariate GF-Fits, Uganda dataset

LC

	POC-CCA	DNA-TaqMan	SEA-ELISA	Kato-Katz1	Kato-Katz2
POC-CCA	0.00				
DNA-TaqMan	0.60	0.00			
SEA-ELISA	10.37	3.48	0.02		
Kato-Katz1	1.66	0.51	0.22	0.00	
Kato-Katz2	0.58	0.13	0.13	2.49	0.00

LCRE

	POC-CCA	DNA-TaqMan	SEA-ELISA	Kato-Katz1	Kato-Katz2
POC-CCA	0.00				
DNA-TaqMan	0.32	0.00			
SEA-ELISA	9.54	3.09	0.00		
Kato-Katz1	0.58	0.16	0.01	0.00	
Kato-Katz2	0.02	0.00	0.12	1.59	0.00

FM

	POC-CCA	DNA-TaqMan	SEA-ELISA	Kato-Katz1	Kato-Katz2
POC-CCA	0.01				
DNA-TaqMan	0.35	0.01			
SEA-ELISA	4.00	3.11	0.06		
Kato-Katz1	1.99	3.14	0.44	0.03	
Kato-Katz2	0.92	2.16	0.07	11.78	0.03
