# Supplementary figures and images for: Latent Class Analysis: Insights about design and analysis of schistosomiasis diagnostic studies
Source: PLoS Negl Trop Dis. 2021 Feb 4;15(2):e0009042. doi: 10.1371/journal.pntd.0009042 (PMC7888681; doi:10.1371/journal.pntd.0009042)

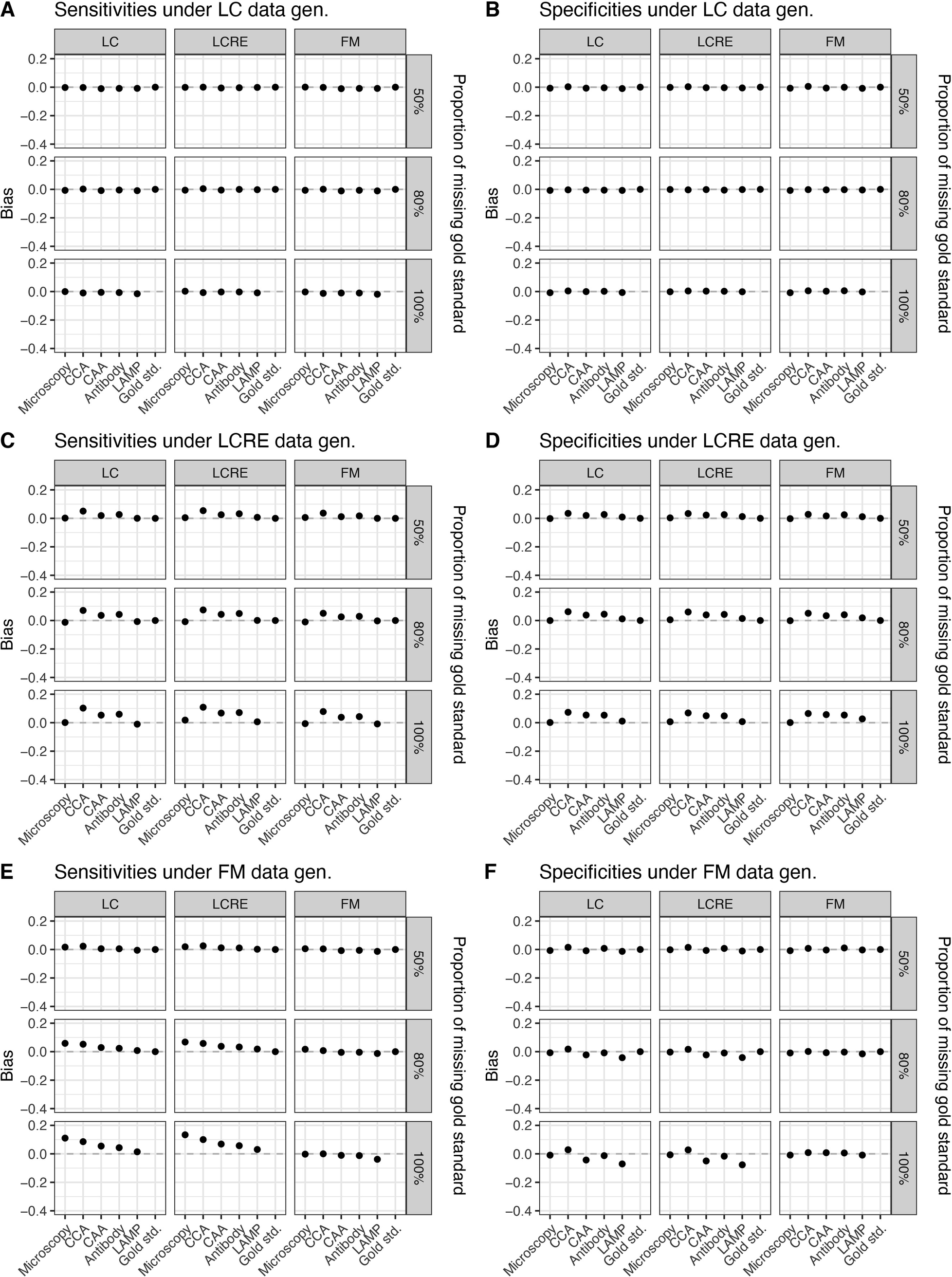

Supplement: S1 Fig — Bias of parameter estimates (sensitivities and specificities) as estimated by the LC, LCRE and FM models under differing proportions of missing gold standard, and under differing data generating mechanisms (sample size = 250, prevalence = 0.4). (TIF) [file pntd.0009042.s002.tif]

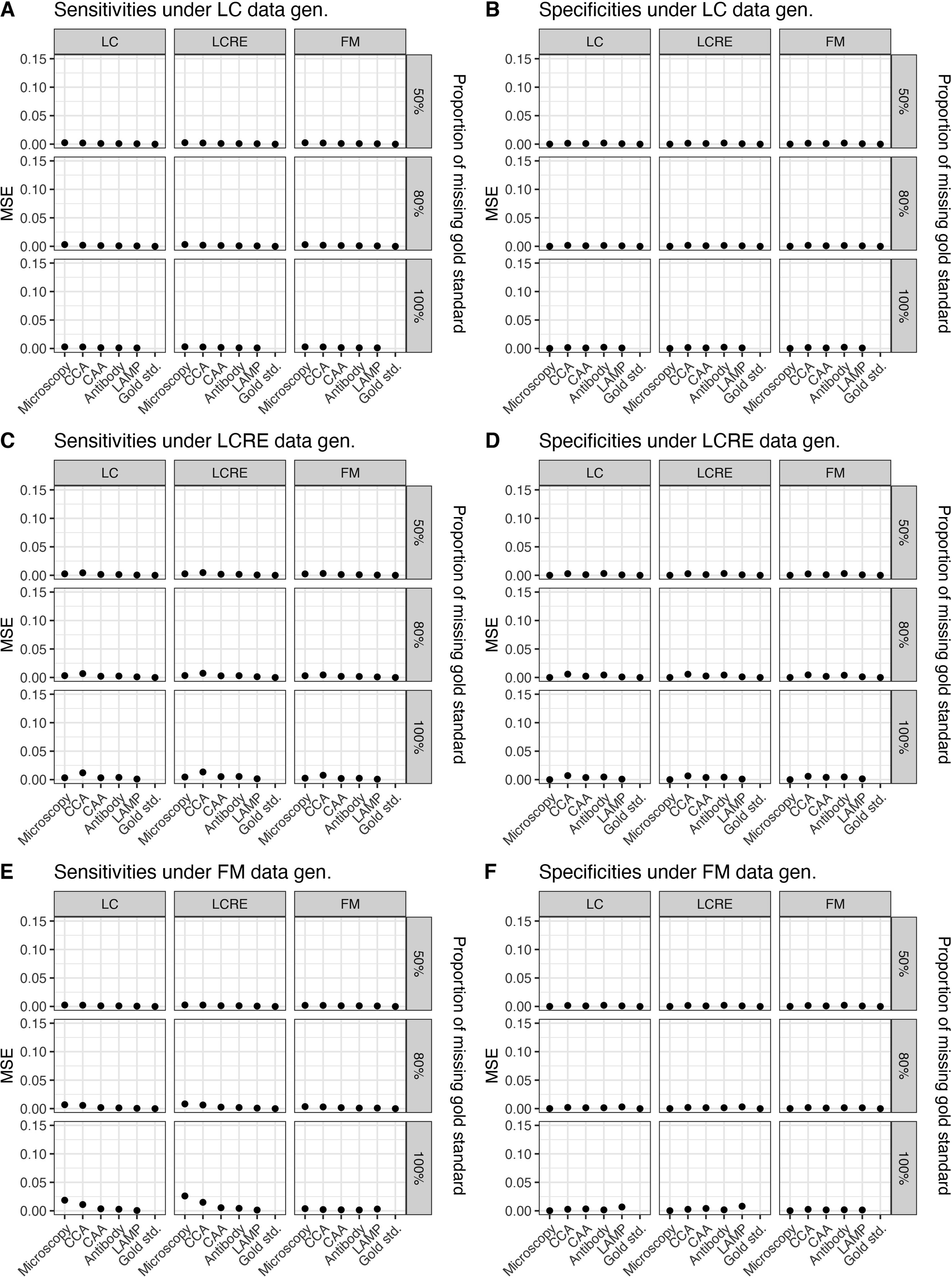

Supplement: S2 Fig — Mean squared error of parameter estimates (sensitivities and specificities) as estimated by the LC, LCRE and FM models under differing proportions of missing gold standard, and under differing data generating mechanisms (sample size = 250, prevalence = 0.4). (TIF) [file pntd.0009042.s003.tif]
