# Supplementary material for: Latent Class Analysis: Insights about design and analysis of schistosomiasis diagnostic studies
Source: PLoS Negl Trop Dis. 2021 Feb 4;15(2):e0009042. doi: 10.1371/journal.pntd.0009042 (PMC7888681; doi:10.1371/journal.pntd.0009042)
Supplement: S1 File — JAGS code for fitting the latent class model under conditional independence, the latent class model with Gaussian random effects, and the finite mixture latent class model. (PDF) [file pntd.0009042.s004.pdf]

S1 File: JAGS code

Note that in the following, class 1 indicates diseased class, and class 2 indicates the healthy class.

Latent Class Model with Conditional Independence

```
model{
for (i in 1:n) {
  d[i] ~ dbern(tau)
  for (j in 1:p) {
    pi.x[i, j] <- d[i] * pi[j,1] + (1 - d[i]) * pi[j,2]
    X[i, j] ~ dbern(pi.x[i, j])
  }
}

# Priors (uninformative)
tau ~ dbeta(1,1)
for (j in 1:(p-1)) {
  pi[j,1] ~ dbeta(1,1)
  pi[j,2] ~ dbeta(1,1)
}

# Gold standard item
pi[p, 1] <- 1  # This fixes sens and spec to 1.If no gold standard
pi[p, 2] <- 1  # then assign dbeta(1,1) priors.

# Sensitivities and specificities
for (j  in 1:p) {
  sens[j] <- pi[j,1]
  spec[j] <- 1 - pi[j,2]
}
}

#data# X, n, p
#monitor# tau, sens, spec, deviance
```

Latent class Gaussian random effects model

```
model{
  for (i in 1:n) {
    d[i] ~ dbern(tau)
    for (j in 1:p) {
      u1[i,j] ~ dnorm(beta[j,1], psi[1])
      u0[i,j] ~ dnorm(beta[j,2], psi[2])
      pi.x[i,j] <- d[i] * phi(u1[i,j]) + (1 - d[i]) * phi(u0[i,j])
      X[i,j] ~ dbern(pi.x[i,j])
    }
  }
```

```
  # Priors
  tau ~ dbeta(1,1)
  for (k in 1:2) {
    psi[k] ~ dgamma(0.1, 0.1)
    sigma[k] <- 1 / sqrt(psi[k])
  }
  for (j in 1:(p-1)) {
    beta[j,1] ~ dnorm(0,0.01)
    beta[j,2] ~ dnorm(0,0.01)
  }

  # Gold standard item
  beta[p,1] <- 100   # This fixes sens and spec to 1. Assign
  beta[p,2] <- -100  # dnorm(0,0.01) prior if no gold std.

  # Sensitivities and specificities
  for (j  in 1:p) {
    sens[j] <- phi(beta[j,1] / sqrt(1 + pow(sigma[1], 2)))
    spec[j] <- 1 - phi(beta[j,2] / sqrt(1 + pow(sigma[2], 2)))
  }
}

#data# X, n, p
#monitor# tau, sens, spec, beta, deviance
```

Finite Mixture Latent Class Model

```
model{
  for (i in 1:n) {  # individuals
    for (j in 1:p) {  # items
      Y[i,j] ~ dbern(A[i,j])
      A[i,j] <- pow(w[j, 1] * d[i] + (1 - w[j,2]) * (1 - d[i]), 1 - l[i])
        - l[i] * (1 - d[i])
    }
    l[i] ~ dbern(pi.l[i])
    pi.l[i] <- d[i] * eta[1] + (1 - d[i]) * eta[2]
    d[i] ~ dbern(tau)
  }

  # Priors
  tau ~ dbeta(1,1)
  eta[1] ~ dbeta(1,1)
  eta[2] ~ dbeta(1,1)
  for (k in 1:2) {
    for (j in 1:(p-1)) {
      w[j,k] ~ dbeta(1,1)
    }

    # Gold standard item
```

```
    w[p,k] <- 1  # if no gold std, assign dbeta(1,1) prior
  }

 # Sensitivities and specificities
  for (j  in 1:p) {
    sens[j] <- eta[1] + (1 - eta[1]) * w[j,1]
    spec[j] <- eta[2] + (1 - eta[2]) * w[j,2]
  }

}

#data# I, J, K, Y
#monitor# tau, sens, spec, eta, deviance
```
