# Supplementary material for: Latent Class Analysis: Insights about design and analysis of schistosomiasis diagnostic studies
Source: PLoS Negl Trop Dis. 2021 Feb 4;15(2):e0009042. doi: 10.1371/journal.pntd.0009042 (PMC7888681; doi:10.1371/journal.pntd.0009042)
Supplement: Attachment — Submitted filename: reviewers.docx [file pntd.0009042.s005.docx]

***Methods
-Are the objectives of the study clearly articulated with a clear testable hypothesis stated?
-Is the study design appropriate to address the stated objectives?
-Is the population clearly described and appropriate for the hypothesis being tested?
-Is the sample size sufficient to ensure adequate power to address the hypothesis being tested?
-Were correct statistical analysis used to support conclusions?
-Are there concerns about ethical or regulatory requirements being met?***

***Reviewer #1: (No Response)

Reviewer #2: The authors proposed to assess the fit of three different types of model to real data - latent class models with the conditional independence assumption met, a random effect latent class model and a finite mixture model, and to use simulated data to explore how improved gold standard measures would affect sensitivity and specificity estimates. Overall the work seems to be very thorough and well thought out. It address an interesting question in a time when epidemiological modelling comes under considerable scrutiny.***

Thank you for your positive comments ***There is no comment on the ethics of the data used. Indeed there is no real methods section that covers the data at all. I think this is quite bold given that the authors state lacking or questionable data as a problem for LCA. It also makes following the manuscript quite difficult.***

The real data analysis of the current paper is secondary analysis of previously published studies. We have added a sentence in the revised manuscript about de-identification of data before release to the researchers and previous consent of study subjects. We have also added a revised methods section as suggested.

***Essentially this paper could provide a valuable toolkit for those with less expertise but wishing to use the correct modelling method. However, the methods are diffuse throughout the paper, making it difficult to follow and hard for these methods to be extracted and replicated (I appreciate that the scripts are available but the text has to complement this). I would recommend making a very clear cut methods section first, that covers the data (real and simulated) and the models and the model selection all in one section. I would also recommend some sort of table or schematic (or combination) that lays out the differences in the three models and the differences in the goodness-of-fit tests.***

Agreed and amended. A methods section first, that covers the data (real and simulated) and the models and the model selection all in one section as well as a table outlining GoF tests has now been added.
 ***On that note, it is difficult to ascertain whether the authors are recommending against the first GOF test due to sparseness, or rather that those using these models should use both GOF tests. Again I think this could be cleared up with more definitive explanations here.***

Agreed and amended. We have revised and recommending both GOF tests to be used.

***From the introduction to the 'results with real data' section is quite long winded and repetitive. By streamlining this the authors would be able to really put a lot more detail into the methods, which seeing as this comes across first and foremost as a methods paper, would make sense.***

Agreed and amended. ***Small methodology side notes:
- I am unsure that 40% prevalence constitutes a truly high prevalence setting in the simulations. Perhaps this is an in-between value for S. mansoni and S. haematobium, but could probably have a small line of explanation.***

Thank you for this insightful comment-we have now clarified that this an in-between value for *S. mansoni* and *S. haematobium*

***- In line 209 the authors state that there are 5 binary variables for both data sets, but the data for S. mansoni has only 4 diagnostic tests so what is the last variable. Again this could be cleared up with some sort of schematic or table, even in the SM.***

When we describe the real data for *S. mansoni* infection we are explaining that we have indeed been using four diagnostic measures. We are also explaining that microscopy of duplicate Kato-Katz smears from two consecutive stools are counting as two observed tests in our statistical analyses. ***Reviewer #3: -***

--------------------

***Results
-Does the analysis presented match the analysis plan?
-Are the results clearly and completely presented?
-Are the figures (Tables, Images) of sufficient quality for clarity?

Reviewer #1: Table 1 is unclear. The authors state that these are tests for Schistosoma mansoni infections. However, it is not clear how dipsticks are relevant. Typically, any dipstick for schistosome infection diagnosis tests for microhematuria, which is only present in persons with Schistosoma haematobium infections. If there is another dipstick for S. mansoni, it should be described. Also, it is not at all clear how they determined the sensitivities and specificities for the different tests--are they hypothetical, best guesses, or available in published literature? Obviously, published literature is best but it must be cited. In addition, as stated elsewhere, the gold standard test for schistosomiasis diagnosis is a mythical beast.***

Thank you for this insightful comment.

There was a typo error in Table 1 which we have now corrected. We fully agree about the previous confusion of dipstick for *S. mansoni* infection which we have now replaced with the Circulating Cathodic Antigen test (CCA) in the revised Table and ShinyApp. We have also included in the revised manuscript that we specified the sensitivities and specificities for the different tests as best guesses.

With regards to the gold standard for schistosomiasis diagnosis certainly, with the African schistosomes, some work has been done to answer in the affirmative, the detection of species specific DNA in body fluids, either blood or urine. Lodh et al (2013) carried out a study in Zambia to compare KK, CCA and DNA (from urine) in an exposed population in Zambia. A total of 89 volunteers were examined, KK showed 45/89 infected, CCA 55/89 (6 false positive) and 79/89 had detectable *S. mansoni* DNA. Clearly if there is a schistosomiasis gold standard, it is the presence of parasite specific DNA. For *S. haematobium* work has been done in Western Nigeria by Ibironke et al. 2012, comparing the presence of parasite eggs detected on the filter paper from the filtered urine, haematuria detected by dipstick and detection of the Dra1 DNA extracted from the filters. Some 400 specimens were examined in that study and the conclusion was that detection of Dra1 fragment is a definitive test for the presence of *S. haematobium* infection.

We have now clarified in the revised version of this ms that the principal aim of the current study is not necessarily to promote or demote particular available diagnostic tests but to show under which scenarios latent class modelling approaches might work or not for the evaluation of existing schistosomiasis diagnostics. Assuming the presence of parasite specific DNA as the schistosomiasis gold standard based on evidence from two previously studies, we are offering methodological solutions for small sample sizes and limited number of diagnostic tests. If the parasite specific DNA cannot be accepted as the schistosomiasis gold standard then the solution for more accurate diagnostic studies are larger sample sizes for high prevalence. Larger number of diagnostic tests (i.e. 10) included in these studies can enable correct identification of the three models when the CI assumption is violated irrespective of sample size.

***Reviewer #2: I do not think the results section delivers the results in a clear, concise manner. There are a lot of methods and discussion dotted through it. For the reader to really make the most of the results this really needs streamlining.***
Agreed and amended.

***Perhaps tables comparing outputs of the models would be helpful, particularly in the real data results section as there is no mention of any figures within the real data results section for readers to refer to. The Shiny App here is helpful, but something visual and immediately available for readers to look at whilst reading would be very helpful.***
Agreed and amended. We have added a table comparing outputs of the models for the real datasets.

***During the simulations results, there is a consistent message that more gold standard observations improve the model outputs in various ways. However, at no point is there a mention of the fact that there isn't a schistosomiasis gold standard. So what this 100% specificity 100% sensitivity gold standard observation is based on in reality, I can not see.***

The simulations show that for small sample sizes (i.e. 250) and small number of included diagnostic tests (i.e. 5) and under violation of the conditional independence assumption, the inclusion of partial gold standard indeed improves the model outputs in various ways, regardless of low or high prevalence (i.e. 8 % or 40 %). However, for assumed prevalence of 40 %, and sample size of 1000 even under violation of conditional independence, the inclusion of gold standard is not that important.

As previously mentioned during our responses to these reviews, we are assuming the presence of parasite specific DNA as the schistosomiasis gold standard based on evidence from two previously studies (Lodh et al. 2013; Ibironke et al. 2012). Based on this assumption, we are exploring and offering methodological solutions for small (i.e. 250) and large (i.e. 1000) sample sizes and limited number of diagnostic tests (i.e. 5). However, if the parasite specific DNA cannot be accepted as the schistosomiasis gold standard, then the solution for more accurate diagnostic studies in general is consideration of larger number of diagnostic tests included in the latent class models, or with limited number of diagnostic tests Bayesian estimation of latent class models with informative priors and/or to consider covariates such as gender and age on the prevalence and the sensitivities and specificities of the diagnostic tests.

***Reviewer #3: The estimated sensitivity of 80% of a single Kato-Katz in the Uganda dataset is inconsistent with the literature while the specificity of POC-CCA is most likely underestimated with 80%. The sensitivity of Kato-Katz is often determined in comparison with multiple Kato-Katz test as the 'gold' standard which has a very high specificity but imperfect sensitivity. Therefore, the sensitivity of a single Kato-Katz test, which generally is in the order of 50% in these studies, is overestimated and can be used as an upper bound. The implausible estimates of the sensitivity of Kato-Katz is indicative of the used models for the conditional dependence not fitting the data generating process well as explained also below.***

Thank you for this insightful comment. We have included these comments in the revised results section.

--------------------

***Conclusions
-Are the conclusions supported by the data presented?
-Are the limitations of analysis clearly described?
-Do the authors discuss how these data can be helpful to advance our understanding of the topic under study?
-Is public health relevance addressed?

Reviewer #1: The conclusions that latent class modeling is not very accurate in the absence of a gold standard is well supported. However, because no gold standard exists, there is not a great deal of public health relevance or a pathway forward to advance understanding of schistosome diagnostic test evaluation.***

Thank you for your comment.

The simulations show that for small sample sizes (i.e. 250) and small number of included diagnostic tests (i.e. 5) and under violation of the conditional independence assumption, the inclusion of partial gold standard indeed improves the model outputs in various ways, regardless of low or high prevalence (i.e. 8 % or 40 %). However, for assumed prevalence of 40 %, and sample size of 1000 even under violation of conditional independence, the inclusion of gold standard is not that important.

As previously mentioned during our responses to these reviews, we are assuming the presence of parasite specific DNA as the schistosomiasis gold standard based on evidence from two previously studies (Lodh et al. 2013; Ibironke et al. 2012). However, if the parasite specific DNA cannot be accepted as the schistosomiasis gold standard, then the solution for more accurate diagnostic studies in general is consideration of larger number of diagnostic tests included in the latent class models, or with limited number of diagnostic tests Bayesian estimation of latent class models with informative priors and/or to consider covariates such as gender and age on the prevalence and the sensitivities and specificities of the diagnostic tests.

In our opinion these are important findings of public health relevance showing what can work and can’t work in schistosomiasis diagnostic evaluation and offering the pathway forward for further relevant and more valid research work.
 ***Reviewer #2: The discussion is clear and well thought out. However, there are bits throughout the results that I think really belong in here. Though the implications for public health are referred to, I do think that overall the authors make many suggestions without any mention of their application in the field. I think that this cross-over is an important consideration in modelling studies and will add weight to the findings, making them more accessible to non-modelling readers. With that said, I am still not sure how the authors address the lack of a gold standard to begin with, if they suggest that this should be included in the model more often. I appreciate that this is somewhat dependent on the infection system in question, and varies between S. haem. and mansoni, but as these are the datasets the authors have used, it deserves a some discussion.***

Thank you for your positive comment. We have revised further discussion section as suggested. We have now clarified that we are assuming the presence of parasite specific DNA as the schistosomiasis gold standard based on evidence from two previously studies (Lodh et al. 2013; Ibironke et al. 2012). However, if the parasite specific DNA cannot be accepted as the schistosomiasis gold standard, then the solution for more accurate diagnostic studies in general is consideration of larger number of diagnostic tests included in the latent class models, or Bayesian estimation of latent class models with informative priors and/or to consider covariates such as gender and age on the prevalence and the sensitivities and specificities of the diagnostic tests.

***There is also no real discussion of the other ways in which more recent modelling studies use LCA and do attempt to address the lack of a gold standard. I would like to see more discussion on how the three models here compare to the current literature.***

Agreed and amended. We have revised discussion accordingly and point to other valid LCA applied diagnostic studies without a gold standard.

--------------------

***Summary and General Comments
Use this section to provide overall comments, discuss strengths/weaknesses of the study, novelty, significance, general execution and scholarship. You may also include additional comments for the author, including concerns about dual publication, research ethics, or publication ethics. If requesting major revision, please articulate the new experiments that are needed.***

***Reviewer #1: The authors' main conclusion, which is probably correct, is that the way latent class analyses have been done when comparing different tests for schistosome infections has severe limitations. However, their solution to this problem, to use a "gold standard" test to evaluate at least a portion of the samples, is in itself problematic. Namely, there is no gold standard test for schistosome infections. If there were a gold standard test, latent class modeling would be unnecessary--the alternative tests could just be compared to the gold standard to determine their sensitivity and specificity. They allude, very briefly, to a couple of molecular assays that "could" be a gold standard for schistosomiasis diagnostics but provide no evidence why these tests are any better than all of the other imperfect tests available. The claims that these tests could be gold standards are only backed up by the authors of the referenced papers.

Thus, while the authors point out what is likely a very real problem with the way analysis of diagnostics is currently done, they really provide no viable solution, making the paper not all that useful. What would be more useful is while acknowledging that no gold standard exists, presenting what might be the least worst alternative study design for diagnostics analysis, including study size, prevalence, and number of tests compared (although this is likely to be limited by what is available as well).***

Thank you for your comment.

However, we do not agree that our paper is not at all useful. To our knowledge, to date there has not been any similar study showing what can work and cannot work with latent class modelling in the field of schistosomiasis and other tropical diseases diagnostic studies. On the other hand, several studies have been already published not having considered the issues the current paper highlights. The current findings can alert researchers and reviewers for future more accurate diagnostic studies in the field.

We have now revised our discussion specifically clarifying that in the scenario of low schistosomiasis prevalence, including 20 % or 50 % of partial gold standard and assuming that this is the parasite specific DNA, this could improve model outputs even in a sample size of 250 participants. If the proposed gold standard is not an acceptable solution, with limited number of diagnostic tests then one should look at other LCA modelling solutions such as covariates or/and Bayesian estimation with informative priors on prevalence and diagnostic performance (i.e. sensitivities and specificities) to maximise the potential of such models for more accurate results.

***The authors should also present their findings in the context of the particular use case for the assay as this can greatly influence the test utility. For example, testing antibody levels can be useful for initial mapping where no previous treatment has been conducted or following presumed interruption of transmission in younger age groups. However, antibody testing is not useful at all to monitor and evaluate an ongoing treatment program in school children.***

Thank you for this comment. As previously mentioned during this review, we have now clarified in the revised version of this ms that the principal aim of the current study is not necessarily to promote or demote particular available diagnostic tests but to show under which scenarios latent class modelling approaches might work or not for the evaluation of existing schistosomiasis diagnostics. We fully though agree that antibody test is useful for schistosomiasis mapping and not treatment. We thus are not commenting further our findings in the context of the particular use case for the assay as we feel that this suggestion is beyond the aims of the current paper.

***Reviewer #2: Overall:

This paper addresses an important topic and could provide a really amazing toolbox. The author is clearly well informed and is passionate about the proper use of the tool and I can totally see why. I also think the Shiny App is a nice way to visualise what they have done, it really helped me to understand what the purpose of the paper was.***

Thank you very much for your positive comments. ***With that said, there is so much information here, that is very diffuse in its presentation, particularly in the introduction. This makes it very difficult to find the purpose of the paper until much later in. The results and the Shiny App therefore don't stand out and the shiny App should not be used as a crutch for results to lean on. Indeed the results are hard to tease out, despite actually being very interesting and quite important to this field.

My greatest suggestion would therefore be to restructure and streamline a lot of the paper.***

Thank you very much for these insightful comments. We have now restructured and streamlined the paper as suggested.

***Some additional comments:

Abstract:

When the authors mention issues of the model when applied to a small number of diagnostics, do this mean a small number of different types of diagnostics (i.e. KK vs, KK & CCA, vs KK, CCA & CAA) or a small number of results from each diagnostic (i.e. sample size)? This becomes clear a little later on, but I’d expand on this slightly here as it is confusing.***

When we refer to small number of diagnostic tests, we mean the number of diagnostic tests included in the LCA (i.e. most often 3-5 diagnostic tests).

***Author Summary:

“the lack of a gold standard due to mainly lack of appropriate equipment and training of technicians across the endemic countries because of scarce financial resources”

This is quite a narrow statement that entirely misses out the variability of certain methods due to biological phenomena that would occur even for the most highly trained technician with the most expensive equipment (i.e. KK egg variability, CCA detecting juveniles etc). It also does not account for the fact that counting worms in a host would be too invasive unless that host is dead.***

Agreed and amended. We have also included in the above sentence the variability of certain methods due to biological phenomena.

***Main Body:

Lines 91-92: This would suggest quite a fundamental flaw in the data collection approach but unlike other paragraphs there is no citation to support that this has indeed happened anywhere. If the authors can find no evidence for it then say this is an additional consideration, or preferably expand this sentence into a paragraph and add an example.***

Agreed and amended. We have clarified that this is an additional consideration.
 ***Line 76: Individuals do not have to be from low middle-income countries be co-infected. Surely individuals in any setting where both infections occur can experience co-infection - I understand they are connected but it is not a pre-requisite.***

Agreed and amended. We have removed the low middle income countries.
 ***Lines 94-121 are hard to follow. The authors give lots of examples and details of models and authors, but I do not think they are tied into a clear progression of the paper. Perhaps a table with the models and a summary of what they achieve or a streamlining of the section that feeds more concisely into what the authors propose to do in the final paragraph of this section, would be easier to follow.

On from the previous comment, in the final line of the paragraph, it sounds like the authors will move into what they propose to do, however your next section brings up types of models mentioned in only parentheses at the top of the previous section. I suggest streamlining these two sections and making clearer the purpose of them in relation to the purpose of the paper. These models presented in the second paragraph are the crux of the work and should be mentioned more thoroughly earlier on. On that note, remove some of the more crucial model description from the SM and put in the main text to aid the reader.***

Agreed and amended. We have incorporated model description and restructured the two sections *‘An alternative solution: Inclusion of partial gold standard’* as well as *‘Statistical models with conditional dependencies’* in the revised ms.

***Line 124: “Albert & Dodd proposed [insertion: a] solution”***

Agreed and amended.

***Lines 126 – 128: Albert & Dodd were talking about radiography and human error, rather than biological variability. It would be helpful here if the authors gave some examples of how this is possible in a setting where the gold standard cannot be acquired because it would be too invasive, or to have another approach for a gold standard that can be conducted in the field. I believe Lamberton et al (2014) would be a good source here.***

As previously mentioned during this review we have now revised and clarified in the new current version of this ms that we are assuming that the schistosomiasis gold standard is the parasite specific DNA from urine. We do not think that this is invasive but if schistosomiasis diagnostics experts think that the combination of other specific schistosomiasis diagnostic tests can yield 100 % sensitivity and specificity, this could act as a partial gold standard in the modelling solution we are exploring and proposing here. We have cited the ‘Lamberton PH, Kabatereine NB, Oguttu DW, Fenwick A, Webster JP. PLoS Negl Trop Dis. 2014 Sep 11;8(9):e3139. doi: 10.1371/journal.pntd.0003139.[Sensitivity and specificity of multiple Kato-Katz thick smears and a circulating cathodic antigen test for Schistosoma mansoni diagnosis pre- and post-repeated-praziquantel treatment’ as proposed.](https://pubmed.ncbi.nlm.nih.gov/25211217/)

***Line 150 – typo “Goodness-of-fit test statistics and measures of fit”***

Agreed and amended.
 ***Lines 355-377: This really is discussion material.***

We have removed some sentences from here and added them to the discussion section. However we still feel that we need a concluding paragraph to close the simulation section.

***Lines 275-276: I do not think the authors mention these parts of the model anywhere else in the paper. They do no not need to be mentioned here, it just adds confusion to readers who have otherwise never been introduced to these models.***

Agreed and amended. We have removed this sentence. ***Reviewer #3: The manuscript is a well written methods paper comparing three ways to incorporate conditional dependence between diagnostic tests using two datasets. It is evaluated whether including an additional gold standard diagnostic test for some of the individuals would improve overall estimation of diagnostic sensitivity and specificity. Results are presented in an innovative way using a shiny app.***

Thank you for your positive comment.
 ***I agree with the general message of the manuscript that having a good diagnostic test among the set of diagnostic tests improves estimation of sensitivity and specificity. However, I am not convinced of the usefulness of the results and used models for the specific case of schistosomiasis for the following reasons:

1. The authors discuss including gold standard diagnostic tests in parallel to improve estimation. However, the sources mentioned do not state that PCR can be considered such a gold (or even silver) standard for schistosomiasis. The source below for example estimates the sensitivity of PCR for schistosomiasis to 69% and the specificity to 91%. It is somewhat self-evident that including a diagnostic test with fully known sensitivity and specificity improves estimation in latent class models. Is the contribution of this paper mostly to quantify that?
Colley, Daniel G., et al. "A five-country evaluation of a point-of-care circulating cathodic antigen urine assay for the prevalence of Schistosoma mansoni." The American journal of tropical medicine and hygiene 88.3 (2013): 426-432.***

The Colley et al. 2013 paper used LCA and through such an analysis indeed this study estimated sensitivity and specificity for the PCR stool assays to be 69% and 91%, respectively. As mentioned previously during this review, we have now clarified that we are assuming the presence of parasite specific DNA from urine as the schistosomiasis gold standard based on evidence from two previously studies (Lodh et al. 2013; Ibironke et al. 2012). Furthermore, the principal aim of the current study is not necessarily to promote or demote particular available diagnostic tests but to show under which scenarios latent class modelling approaches might work or not for the evaluation of existing schistosomiasis diagnostics.

If the parasite specific DNA from urine cannot be accepted as the schistosomiasis gold standard, we provide other recommendations for more accurate diagnostic studies in general. More precisely we recommend consideration of larger number of diagnostic tests (i.e. >=10) included in the study design and the latent class models. With limited number of diagnostic tests, we recommend Bayesian estimation of latent class models with informative priors and/or to consider covariates in LCA such as gender and age on the prevalence and the sensitivities and specificities of the diagnostic tests. In addition, the current study showed that for assumed prevalence of 40 %, and sample size of 1000 even under violation of conditional independence, the inclusion of gold standard is not that important in the LCA or LCRE or finite mixture models. Since several studies have been already published not having considered the issues the current paper highlights, our study is valuable alerting researchers and reviewers for future more accurate diagnostic studies in the field.

***2. The models models used to incorporate conditional dependence between diagnostic tests do not match the data generating process for schistosomiasis particularly well. For example the paper ignores the effect of infection intensity on diagnostic sensitivity (except one remark on line 85 as one effect among many) which is a if not the major driver of conditional dependence between different diagnostic tests. Schistosomiasis is not like many other conditions a binary status of either having the disease or not but diagnostic tests and morbidity depend on the number of worm-pairs harboured by an individual. Many studies have shown that the sensitivity changes strongly with infection intensity for example for both Kato-Katz and POC-CCA the relation is also given in the paper by Colley et al. in the reference above. There have been examples of applications of latent lass models with random effect structures inspired by what is known about the data generating process of
schistososomiasis that are not at all discussed in this paper and that could be used to create more realistic simulations (see references below). Therefore, I doubt how relevant the simulation results are for the specific case of schistosomiasis.
Prada, Joaquín M., et al. "Understanding the relationship between egg-and antigen-based diagnostics of Schistosoma mansoni infection pre-and post-treatment in Uganda." Parasites & vectors 11.1 (2018): 21.
Bärenbold, Oliver, et al. "Translating preventive chemotherapy prevalence thresholds for Schistosoma mansoni from the Kato-Katz technique into the point-of-care circulating cathodic antigen diagnostic test." PLoS neglected tropical diseases 12.12 (2018): e0006941.***

Thank you for pointing us to these two important studies studying the relationship between the two most commonly used diagnostic tools for S. mansoni infection. The aim of our paper was to explore under which scenarios latent class models for different prevalence might work or not and what solutions might exist to improve model fits and provide more accurate results. The existing simulations have required significant time and effort to complete and present. We believe that these simulations provide very useful results, recommendations and tools for future studies. We are now citing the two proposed studies as evidence to refine these simulations if one wished to investigate this matter further, although we are confident that the main conclusions about small sample sizes and the inclusion of partial gold standard would not change. We are providing R codes for other researchers to repeat such analyses in their studies. We are also happy to share codes for simulations if other researchers were interested in this kind of research and we have included this in the revised version of our ms.

***3. There are only two small datasets used in this study to attempt to make a selection between the three proposed models. One with 220 and the other with 258 individuals and combined maybe about 160 positive individuals. I don't think that it is possible to select the most suitable model with that little data, especially when the models are fit to the two datasets separately. This could for example be tested by simulating datasets of different sizes from each model and checking how often the correct model will be selected with the used methodology. To increase power I suggest using for example the openly available data from the paper by Bärenbold et al. at*** [***https://doi.org/10.1371/journal.pntd.0006941.s002***](https://protect-eu.mimecast.com/s/K9Q_Cj814Cnkm82uxsgPe?domain=doi.org) ***with up to 12 diagnostic tests from many different sites and in total several thousand individuals or the dataset used in the paper by Prada et al. at in 'additional file 3' at*** [***https://doi.org/10.1186/s13071-017-2580-z***](https://protect-eu.mimecast.com/s/MRuJCk714TOZB97h0oGvs?domain=doi.org)***.***

Thank you for your comment. Our simulations highlighted the fit of LC, LCRE and FM models was poor across the board for small sample sizes when data generating models were complex. Through the real datasets analysis, we also provided recommendations how to correctly interpret the results from the three models for these small sample sizes.

We have now clarified that the aim of the current paper is to show how LCMs and two altered versions of these models might work or not, in small sample sizes with a few number of diagnostic tests (i.e. 5) and at differing levels of prevalence. We also perform the same simulations for a larger sample size of 1000, which we use to demonstrate potential improvements solely due to a larger sample. The issue of including more diagnostic tests in latent class models is a very interesting one but this is beyond the aim of the current paper. In additional it has already been investigated by Albert and Dodd 2004 suggesting that we can indeed correctly identify the most suitable model. As mentioned before we are very happy to share with any interested researchers the codes for simulations upon request. Finally, we have recommended the suggested citations and available datasets for other researchers to apply the proposed models in this study.
