# Supplementary material for: Latent Class Analysis: Insights about design and analysis of schistosomiasis diagnostic studies
Source: PLoS Negl Trop Dis. 2021 Feb 4;15(2):e0009042. doi: 10.1371/journal.pntd.0009042 (PMC7888681; doi:10.1371/journal.pntd.0009042)
Supplement: Attachment — Submitted filename: Nov20Reviewerresp.docx [file pntd.0009042.s006.docx]

**Reviewer's Responses to Questions and Authors Responses to Reviewers Questions**

Key Review Criteria Required for Acceptance?
As you describe the new analyses required for acceptance, please consider the following:

Methods
-Are the objectives of the study clearly articulated with a clear testable hypothesis stated?
-Is the study design appropriate to address the stated objectives?
-Is the population clearly described and appropriate for the hypothesis being tested?
-Is the sample size sufficient to ensure adequate power to address the hypothesis being tested?
-Were correct statistical analysis used to support conclusions?
-Are there concerns about ethical or regulatory requirements being met?

Reviewer #2: (No Response)

Reviewer #3: -Latent class Gaussian random effects model:
A gaussian random effect is included in each latent class to model dependence between different diagnostic tests. However, there is no diagnostic test specific parameter that determines the influence of the random effect on the sensitivity/specific. In particular I don't see how this model captures the situation where some diagnostic tests are correlated and some are not. This is not solved by the Finite mixture latent class model either.

*In all model specifications discussed in the paper, the diagnostic tests are expected to be correlated since they aim to measure the same latent construct (e.g. status of infection). We have examined latent class Gaussian random effects where the random effect is added in all diagnostic tests as a common effect. Although such models do not specify (in)dependence among certain diagnostic tests, we would expect them to capture such structures when these models provide good fit to the data overall. The individual specific random effect component captures diagnostic tests’ dependencies that are beyond the ones explained by the latent classes. The sensitivities and the specificities computed after equation (2) depend on the variance of the random effect which is allowed to be different in each class but common for all diagnostic tests.*

*If one wanted, a random effect could be added to specific diagnostic tests but that would imply that we have pre-knowledge on which diagnostic tests are correlated beyond the correlation that can be explained by the latent structure.*

*The finite mixture model is proposed to solve the problem of an excessive number of zero and one responses. Ignoring that excess in the data can affect the true dimensionality of the data detected by standard goodness of fit tests and can also distort the parameter estimates and as a result sensitivities and specificities. We have added these new clarifications in the section ‘Statistical models with conditional dependencies’.*

-Goodness-of-fit test statistics measures of fit:
It is reported that the number of events in the two real datasets is too small for the Chi-squared test. This should indicate that more data is needed to evaluate whether any of the models sufficiently fit the latent correlation structure of the data.

*The sparseness problem is also an issue with the increase of the number of diagnostic tests s even if the sample size gets larger. One could perform a parametric bootstrapping to empirically study the distribution of the overall chi-square test statistic. For the models discussed here and for a reasonable number of diagnostic testss and latent classes it will be a feasible task. In the paper, we compliment the overall goodness-of-fit test statistic with information on bivariate residuals. Bivariate residuals have the advantage that they are able to detect pairs of diagnostic tests where the misfit occurs.*

*Taking into account the Reviewer’s comment, we also now conducted a parametric bootstrapping for finding the empirical distribution of the chi-square test statistic and the corresponding p-values. We have now added those results in the revised paper.*

*For each model, we generated 10000 data sets from using the estimated model parameters from the data as the true ones. For each generated data set we computed the chi-square test statistic. To obtain the empirical p-value we count the number of times the test statistic from the original data is bigger than the generated chi-square test statistics.*

*The results are as follows:*

*For the Ghana data set and the three models the values are: LC: X^2=16.42, p=0.982; LCRE: X^2=17.08, p=0.965 and FM: X^2=16.60, p=0.982.*

*For the Uganda data set and the three models the values are: LC: X^2=43.51, p=0.125; LCRE: X^2=47.032, p=0.104; FM: X^2=50.6, p=0.060.*

*The empirical p-values for the Ghana data set indicate a good fit which was already the case by the overall goodness-of-fit test statistic but also the bivariate residuals.*

*In the Uganda data set, the empirical p-values now suggest that all 3 models are a good fit to the data. This result is in line with the fact that the bivariate margins did not detect any misfits in the bivariate residuals.*

*We have added these new calculations in the section ‘Results from real datasets’*

-Inclusion of partial gold standard:
Reference 5 only states that DNA detection is better than existing ones but not that it is a gold standard. Reference 6 is not about schistosomiasis in particular and does not report diagnostic accuracy. Reference 7 is a study in 89 people with Kato-Katz, CCA, and DNA detection which is certainly not sufficient to establish a gold standard. Reference 8 reports 100% sensitivity and specificity without confidence intervals from a dataset with urine filtration, dipstick, and DNA. It reports very low specificity for dipstick probably because all dipstick positive but DNA negative are treated as false-positives. I don't think this supports DNA as a gold standard with any confidence as I can't see how these estimates could be determined. Therefore, the current lack of gold standard used is not due to lack of equipment as stated in the author summary but according to current knowledge due to no gold standard existing. The authors argue that in absence of a gold standard themanuscript is still useful to discuss what models should be used and when to be cautious.

*We have revised our statement in the introduction section to address the reservations of the reviewer as follows: ‘Currently there is no agreed gold standard diagnostic test for schistosomiasis; however we are assuming that in due course the presence of parasite specific DNA in the urine will be acceptable as the gold standard. This is based on evidence from two previously published studies [7, 8] and some more recent research by Archer et al.in Molecules September, 2020. doi: 10.3390/molecules25184175’. We have also revised accordingly the Authors summary.*

*Our manuscript is useful as it provides strong evidence of how the examined latent class models can or cannot provide reliable estimates in different prevalence settings with smaller and larger sample sizes with and without the inclusion of a gold standard. Some of the results are positive and some less so - nevertheless this study still shows to researchers where they can or they cannot look for a solution, and to our opinion this is important for real progress of accurate evaluation of schistosomiasis diagnostics. The aim of the current study is not necessarily to promote or demote particular available schistosomiasis diagnostic tests.*

Reviewer #4: Please see general comments

--------------------

Results
-Does the analysis presented match the analysis plan?
-Are the results clearly and completely presented?
-Are the figures (Tables, Images) of sufficient quality for clarity?

Reviewer #2: (No Response)

Reviewer #3: Results from real datasets:
-Table 1: Sensitivity of eggs is probably overestimated and specificity of ELISA and Circ. Antigens underestimated. I'm surprised that the LCRE model does not change estimates for antigen based tests which are probably heavily correlated but given the model allows only for dependence between all tests or none this might not be a surprise.

*In all model specifications discussed in the paper, the tests are expected to be correlated since they aim to measure the same latent construct (e.g. status of infection). When the correlation among the diagnostic tests is explained by the latent classes there is no need to add the random effect. If there are dependencies among the tests that are not captured by the latent classes, the latent class Gaussian random effects models would be expected to capture those and provide a good fit to the data overall.*

*We have now added these new clarifications in the sections ‘Statistical models with conditional dependencies’****.***

-Table 2: How come the FM models underestimates the 00000 and 11111? I thought point masses are included for those options and should therefore match the data?

*The Reviewer has made a very good point that we now clarify in a footnote under Tables 2 and 4. The expected frequencies that appear in Tables 2 and 4 for those patterns (00000 and 11111) are based on the non-deterministic component of the model. The model has four classes in total, two deterministic and two non-deterministic classes. Given that a respondent is in the ‘zero’ or ‘one’ class there is probability 1 that they will respond 0 or 1 respectively. When we add the expected frequencies taking into account both the deterministic and non-deterministic component for the all zero and all one patterns then the sum gives the total number of observed all zero and all one responses.*

-Table 3: A sensitivity of 79% of Kato-Katz and a specificity of 55% for a DNA based method are not realistic values.

*We do not agree with this observation. The estimated egg sensitivities concern microscopy of duplicate Kato-Katz smears from two consecutive stools (these are counting as two observed tests in the analyses) and as such we think they are quite reasonable. We also provide credible intervals at the end of this revision and in the Shiny App.*

-Table 4: The occurrence of 00000 and 11111 are again underestimated suggesting that the correlation structure is not captured by the model. I still don't understand how the FM model is not able to capture it.

*Please see our response above regarding Table 2.*

-It is reported that for mansoni the fit is worse which could just reflect that the prevalence is higher and therefore there is more power to see discrepancies between the data and the models.

*The fit is worse with respect to the overall goodness of fit test statistic. However, the bivariate residuals do not appear to detect any pair fit violations except for the pair of Kato-Katz 1 and Kato-Katz 2 which is expected and for which both the LC and LCRE show an adequate fit but not the FM model.*

- what are the estimates for the parameters describing the structure violating the conditional independence in the more complex models?

*We are assuming that the reviewer refers to the estimated variances of the random effects.*

*For the Ghana example, the estimates for sigma1 and sigma2 in the LCRE model are 2.25 (95% CI [0.12, 6.35]) and 2.42 (95% CI [0.74, 4.53]) respectively. Whereas in the Uganda example, the estimates for sigma1 and sigma2 in the LCRE model are 4.32 (95% CI [1.13, 7.91]) and 1.63 (95% CI [0.18, 3.63]) respectively. In both cases, the significance of the variances of the random effects suggest a violation of the conditional independence of the test items.*

*We have added a table of parameter estimates for all models for both data examples at the end of this review as well as to the Shiny app in the revised version of the paper.*

-The results indicate that none of the models fits the data well enough but the sample size is too small to know. I'd be interested in a paper putting the models to a severe test to evaluate if any of them is useful to simulate data for schistosomiasis which is why I suggested using larger datasets.

*This is not entirely true. All three models fit the Ghana data adequately. Also in the Uganda data set although the overall chi-square test rejected all three models, the bivariate residuals did not detect any ‘offensive’ items. Furthermore, the empirical p-values obtained from the parameteric bootstrapping indicate a good fit across all models for both data sets.*

Designs of the simulations:
- The sensitivity and specificity values chosen for the simulation do not seem reasonable to me and there are no references given to support the author's 'best guesses'.

*We have added references and clarified that guesses are based on expert knowledge by Professor Clive Shiff.*

- what do the chosen values for sigma in the simulation mean for the correlation between tests? How is it incorporated that not all tests are correlated with each of the other? How are the values specific for schistosomiasis given the reference mentioned?

*In all model specifications discussed in the paper, the tests are expected to be correlated since they aim to measure the same latent construct (e.g. status of infection). The individual specific random effect component captures diagnostic tests’ dependencies that are beyond the ones explained by the latent classes. In the simulation study, by generating data from a LCRE model it implies that the correlations among the tests will be captured by the latent classes but also the random effects. Given a latent class, the diagnostic tests will still be correlated based on the random effect and the higher the variance of the random effect the higher the correlation between the diagnostic tests from the* *same class. If the data have been generated by a LCRE model, a simple LC model will be inadequate to explain the dependencies because more latent structure is needed to account for them. In a real application, one hopes that when those dependencies are present, to be captured by the additional random effect added to the latent class model. In other words, although latent class Gaussian random effects models do not specify (in)dependence among certain tests, we would expect them to capture such structures when these models provide good fit to the data overall. The larger the values of sigma the higher will be the proportion of correlation between the diagnostic tests explained by the random effect.*

*We have now added these new clarifications in the sections ‘Statistical models with conditional dependencies’* *and ‘Design of the simulations’.*

Reviewer #4: Please see general comments

--------------------

Conclusions
-Are the conclusions supported by the data presented?
-Are the limitations of analysis clearly described?
-Do the authors discuss how these data can be helpful to advance our understanding of the topic under study?
-Is public health relevance addressed?

Reviewer #2: (No Response)

Reviewer #3: (No Response)

Reviewer #4: Please see general comments

--------------------

Editorial and Data Presentation Modifications?
Use this section for editorial suggestions as well as relatively minor modifications of existing data that would enhance clarity. If the only modifications needed are minor and/or editorial, you may wish to recommend “Minor Revision” or “Accept”. 

Reviewer #2: (No Response)

Reviewer #3: (No Response)

Reviewer #4: (No Response)

--------------------

Summary and General Comments
Use this section to provide overall comments, discuss strengths/weaknesses of the study, novelty, significance, general execution and scholarship. You may also include additional comments for the author, including concerns about dual publication, research ethics, or publication ethics. If requesting major revision, please articulate the new experiments that are needed.

Reviewer #2: I have only three very minor edits, otherwise I am happy with the revision of this paper. The authors have taken the time to address all the comments and have done so thoroughly. It reads much more smoothly and I really appreciate the more apparent link to field study design.

*Thank you for your positive comment.*

My three comments are: 

1. In the author summary, perhaps using numbers instead of semi-colons to separate out the list of study design issues. 
*Agreed and amended.*

2. Line 49 I think should say "partial gold standard" instead of partially

*Agreed and amended.*

3. Line 52 I think should say "two previous studies" not two previously studies.
*Agreed and amended.*

Reviewer #3: - I do not believe that the corrections sufficiently addresses the major problems previously identified by multiple reviewers.
- There does not seem to be a gold standard diagnostic test for schistosomiasis and this is still one of the major assumptions behind the manuscript.

*We recognize that currently there is no agreed gold standard diagnostic test for schistosomiasis; we are assuming that in due course the presence of parasite specific DNA in the urine will be acceptable as the gold standard. Another practical solution for including partially another ‘gold standard’ could be to combine information on a subset of individuals from different tests and compare those to data from other imperfect diagnostic tests on all individuals via the proposed models. If one would not like to consider inclusion of a ‘gold standard’ this study still provides insights of when such models could provide reliable estimates.*

- The used statistical models do not capture the real correlation structure between diagnostic tests for schistosomiasis and are not validated because the sample size is too small.

*In the paper, we compliment the overall goodness-of-fit test statistic with information on bivariate residuals. Bivariate residuals have the advantage that they are able to detect pairs of items where the misfit occurs. Taking into account the Reviewer’s comment we also now conducted a parametric bootstrapping for finding the empirical distribution of the chi-square test statistic and the corresponding p-values. We have now added those results in the revised paper.*

*For each model, we generated 10000 data sets from using the estimated model parameters from the data as the true ones. For each generated data set we computed the chi-square test statistic. To obtain the empirical p-value we count the number of times the test statistic from the original data is bigger than the generated chi-square test statistics.*

*The results are as follows:*

*For the Ghana data set and the three models the values are: LC: X^2=16.42, p=0.982; LCRE: X^2=17.08, p=0.965 and FM: X^2=16.60, p=0.982.*

*For the Uganda data set and the three models the values are: LC: X^2=43.51, p=0.125; LCRE: X^2=47.032, p=0.104; FM: X^2=50.6, p=0.060.*

*The empirical p-values for the Ghana data set indicate a good fit which was already the case by the overall goodness-of-fit test statistic but also the bivariate residuals.*

*In the Uganda data set, the empirical p-values now suggest that all 3 models are a good fit to the data. This result is in line with the fact that the bivariate margins did not detect any misfits in the bivariate residuals.*

*We have added these new calculations in the section ‘Results from real datasets’*

- Therefore, I do not have confidence that the simulation and conclusions from it are supported for schistosomiasis.
-250 people might be a typical size of a study to determine prevalence but it is simply too low to determine which is the appropriate model to use and to determine diagnostic accuracy of multiple diagnostic tests including their correlation structure.

*This is not entirely true. All three models fit the Ghana data adequately. Also in the Uganda data set although the overall chi-square test rejected all three models, the bivariate residuals did not detect any ‘offensive’ items. As 250 is a similar to a typical sample size in practice to date, such simulations are still useful because they can enhance the evaluations of the examined models for this sample size and provide useful insights into more adequate future data collection. As mentioned before, this study aims to provide guidance to researchers where they can or they cannot look for a solution, and to our opinion this is important for real progress of accurate evaluation of schistosomiasis diagnostics.*

Reviewer #4: In this manuscript, the authors describe and implement three latent class models, using two datasets (one of S. haematobium and one of S. Mansoni). The authors then discuss the limitations of such models to estimate disease prevalence and the issues with the different diagnostics.

I thoroughly enjoyed reading this manuscript, it is mostly clear and well written. The methods are appropriate and highlight some of the challenges of dealing with multiple diagnostics in the absence of a gold standard.

*Thank you very much for your positive comments*
Overall, I feel this manuscript is almost ready for publication, I would like some points clarified further before this is acceptable for publication:

1. I feel the title can be misinterpreted. When I read it, I thought this manuscript was about issues with schisto studies, but really it is issues with Latent class models in general, using a schisto example to discuss it. Most recent schisto diagnostic studies (which are cited in the manuscript), do not use any of the three models described here. I would recommend the authors to consider an alternative title.

*Agreed and amended. We have changed the title to*

*[‘Latent Class Analysis: Insights about design and analysis of schistosomiasis diagnostic studies’](https://journals.sagepub.com/doi/abs/10.1177/0095798420930932)*

2. Following from the above, recent papers on schisto diagnostics use mechanistic or semi-mechanistic approaches, I would like to hear the thoughts of the authors in their discussion as the issues they highlight here could potentially be also in those models (or not)?

*Agreed and amended. We have included in the revised discussion the following sentence: In the current study, we applied data-driven modeling approaches searching for relationships in the available data and accounting for measurement error and dependencies between the diagnostic tests as well as excessive number of zero and one responses, aiming for accurate parameter inference. The latter is a key component for correct predictions from mechanistic approaches, which incorporate the available knowledge of the system into the model. Combination of data-driven and mathematical modeling approaches could offer great synergies with regards to the understanding of interactions between population epidemiology and diagnostic tests characteristics.*

3. I would like the use of binary data discussed a little bit too, many diagnostics (specially in schisto) are not black/white. There is a lot of information that is potentially lost when making them binary (i.e. KK can give intensity, CCA is also somewhat semi-quantitative).

*Agreed and amended. We have included in the revised discussion the following sentence and also added two relevant references: ‘Latent class models can be fitted to binary but also other type of indicators such as continuous, ordinal or mixed. Sensitivities and specificities would need to be carefully considered for those type of data too but in principle the examined models of the current study can be extended to handle any type of data’.*

4. As the main purpose of the paper is to explain these Latent class methods, I worry a bit that the methods section itself is a bit too technical for a majority of the plos NTD audience. I’m not suggesting for the equations to be removed, but I leave it at the authors discretion if they feel some of the methodology text can be clarified further.

*Thank you for this comment. We have now clarified issues about the LCRE and FM in sections* *Results from real datasets, ‘Statistical models with conditional dependencies’ and ‘Design of the simulations’.*

5. Following on the above, I want to commend the authors for the shiny App. I think it is a great addition to the paper and I take my hat off. It is a simple, yet effective way of getting the point across and I know they can be a bit fiddly to code.

*Thank you very much for your positive comment and recognizing the amount of work that exists in the current Shiny App.*

6. Regarding the gold standard. It would be good if the assumption of a gold standard is discussed a bit more. What are the biases of assuming a diagnostic as a gold standard when it is not, as no diagnostic is perfect?

*In all our examples we assumed that the gold standard behaves like a gold standard. This insightful comment will involve another set of simulations in which we allow the gold standard not to have 1 and 0 sensitivity and specificity but close to them. Although this is a very interesting suggestion, it is out of our scope and we have recommended it as a possible investigation to be included in further research.*

7. I was a bit surprised by the result that egg sensitivity was higher in the lower prevalence setting (Tables 1 & 3), as we would expect the opposite, a lower sensitivity with low prevalence. It has to be due to a difference between haematobium and mansoni, so potentially worth a sentence or two?

*We comment on the ‘Results from real datasets’ section that for the Ghanaian dataset (S.haematobium infection), sensitivity estimates for all diagnostic tests, except for ELISA, have very high associated uncertainty and thus no reliable inference can be available for these diagnostic tests sensitivities (including microscopy). In addition, for the Ugandan dataset (S. mansoni infection) the egg sensitivities concern microscopy of duplicate Kato-Katz smears from two consecutive stools (these are counting as two observed tests in the analyses) and as such we think they are quite reasonable. Thus, we have not commented further on suggested difference between the two schistosome species.*

Additional thoughts:
Line 15. Where do the 6 false positives come from?

Line 51. What if DNA is not available? Does DNA necessarily imply live worms (for example after treatment)?

*In the Nigeria work by Lodh et al. (Ref 7) it was demonstrated  that parasite specific DNA is not detectable after two weeks post therapy.*

--------------------

**Parameter estimates for real data models**

Ghana data set (test items used: 1-ELISA, 2-Ultrasound, 3-Haematuria, 4-Eggs, 5-Circ. Antigens)

Latent Class Model

|  | Lower95 | Mean | Upper95 | SD |
| --- | --- | --- | --- | --- |
| prevalence | 0.09 | 0.15 | 0.23 | 0.04 |
| sens[1] | 0.86 | 0.95 | 1.00 | 0.04 |
| sens[2] | 0.46 | 0.63 | 0.83 | 0.10 |
| sens[3] | 0.52 | 0.74 | 0.94 | 0.10 |
| sens[4] | 0.64 | 0.85 | 1.00 | 0.11 |
| sens[5] | 0.30 | 0.48 | 0.66 | 0.09 |
| spec[1] | 0.30 | 0.37 | 0.44 | 0.04 |
| spec[2] | 0.67 | 0.74 | 0.80 | 0.03 |
| spec[3] | 0.82 | 0.87 | 0.93 | 0.03 |
| spec[4] | 0.93 | 0.97 | 1.00 | 0.02 |
| spec[5] | 0.49 | 0.56 | 0.64 | 0.04 |

Latent Class RE Model (class 1 = diseased, class 2 = healthy)

|  | Lower95 | Mean | Upper95 | SD |
| --- | --- | --- | --- | --- |
| prevalence | 0.08 | 0.14 | 0.22 | 0.04 |
| sens[1] | 0.93 | 0.98 | 1.00 | 0.02 |
| sens[2] | 0.45 | 0.65 | 0.83 | 0.10 |
| sens[3] | 0.59 | 0.77 | 0.99 | 0.12 |
| sens[4] | 0.74 | 0.91 | 1.00 | 0.09 |
| sens[5] | 0.28 | 0.46 | 0.66 | 0.10 |
| spec[1] | 0.29 | 0.36 | 0.44 | 0.04 |
| spec[2] | 0.67 | 0.74 | 0.80 | 0.03 |
| spec[3] | 0.81 | 0.87 | 0.92 | 0.03 |
| spec[4] | 0.93 | 0.97 | 1.00 | 0.02 |
| spec[5] | 0.49 | 0.57 | 0.64 | 0.04 |
| beta[1,1] | 1.46 | 6.82 | 12.97 | 3.90 |
| beta[2,1] | -0.35 | 0.91 | 3.02 | 0.85 |
| beta[3,1] | 0.13 | 1.80 | 4.15 | 1.11 |
| beta[4,1] | 0.48 | 4.39 | 12.14 | 3.72 |
| beta[5,1] | -1.68 | -0.19 | 1.16 | 0.65 |
| beta[1,2] | 0.25 | 0.93 | 1.76 | 0.42 |
| beta[2,2] | -2.93 | -1.68 | -0.59 | 0.65 |
| beta[3,2] | -5.14 | -3.00 | -1.06 | 1.11 |
| beta[4,2] | -8.33 | -5.15 | -2.08 | 1.71 |
| beta[5,2] | -1.06 | -0.44 | 0.09 | 0.31 |
| sigma[1] | 0.12 | 2.25 | 6.35 | 2.04 |
| sigma[2] | 0.74 | 2.42 | 4.53 | 1.02 |

Finite Mixture Model (class 1 = diseased, class 2 = healthy)

|  | Lower95 | Mean | Upper95 | SD |
| --- | --- | --- | --- | --- |
| prevalence | 0.07 | 0.15 | 0.24 | 0.05 |
| sens[1] | 0.83 | 0.93 | 1.00 | 0.05 |
| sens[2] | 0.42 | 0.64 | 0.83 | 0.11 |
| sens[3] | 0.59 | 0.78 | 1.00 | 0.11 |
| sens[4] | 0.59 | 0.84 | 1.00 | 0.13 |
| sens[5] | 0.28 | 0.47 | 0.66 | 0.10 |
| spec[1] | 0.30 | 0.37 | 0.44 | 0.04 |
| spec[2] | 0.67 | 0.73 | 0.80 | 0.03 |
| spec[3] | 0.81 | 0.88 | 0.93 | 0.03 |
| spec[4] | 0.92 | 0.96 | 1.00 | 0.02 |
| spec[5] | 0.49 | 0.56 | 0.64 | 0.04 |
| w[1,1] | 0.25 | 0.33 | 0.41 | 0.04 |
| w[2,1] | 0.65 | 0.72 | 0.79 | 0.04 |
| w[3,1] | 0.80 | 0.87 | 0.93 | 0.03 |
| w[4,1] | 0.91 | 0.96 | 1.00 | 0.03 |
| w[5,1] | 0.45 | 0.54 | 0.61 | 0.04 |
| w[1,2] | 0.79 | 0.92 | 1.00 | 0.07 |
| w[2,2] | 0.33 | 0.55 | 0.80 | 0.12 |
| w[3,2] | 0.51 | 0.73 | 0.99 | 0.13 |
| w[4,2] | 0.52 | 0.80 | 1.00 | 0.15 |
| w[5,2] | 0.07 | 0.33 | 0.58 | 0.13 |
| eta[1] | 0.00 | 0.05 | 0.11 | 0.03 |
| eta[2] | 0.00 | 0.19 | 0.39 | 0.11 |

Uganda data set (test items used: 1-POC-CCA, 2-SEA-ELISA, 3-DNA-TaqMan, 4-Kato-Katz1, 5-Kato-Katz2)

Latent Class Model

|  | Lower95 | Mean | Upper95 | SD |
| --- | --- | --- | --- | --- |
| prevalence | 0.41 | 0.47 | 0.54 | 0.03 |
| sens[1] | 0.95 | 0.98 | 1.00 | 0.01 |
| sens[2] | 0.95 | 0.98 | 1.00 | 0.02 |
| sens[3] | 0.87 | 0.92 | 0.96 | 0.03 |
| sens[4] | 0.70 | 0.79 | 0.87 | 0.04 |
| sens[5] | 0.71 | 0.79 | 0.88 | 0.04 |
| spec[1] | 0.72 | 0.80 | 0.88 | 0.04 |
| spec[2] | 0.38 | 0.46 | 0.55 | 0.04 |
| spec[3] | 0.46 | 0.55 | 0.63 | 0.04 |
| spec[4] | 0.97 | 0.99 | 1.00 | 0.01 |
| spec[5] | 0.97 | 0.99 | 1.00 | 0.01 |

Latent Class RE Model (class 1 = diseased, class 2 = healthy)

|  | Lower95 | Mean | Upper95 | SD |
| --- | --- | --- | --- | --- |
| prevalence | 0.41 | 0.48 | 0.55 | 0.03 |
| sens[1] | 0.95 | 0.98 | 1.00 | 0.01 |
| sens[2] | 0.96 | 0.98 | 1.00 | 0.01 |
| sens[3] | 0.87 | 0.92 | 0.96 | 0.03 |
| sens[4] | 0.70 | 0.79 | 0.87 | 0.04 |
| sens[5] | 0.70 | 0.79 | 0.87 | 0.04 |
| spec[1] | 0.74 | 0.81 | 0.89 | 0.04 |
| spec[2] | 0.38 | 0.46 | 0.55 | 0.04 |
| spec[3] | 0.47 | 0.55 | 0.64 | 0.05 |
| spec[4] | 0.99 | 1.00 | 1.00 | 0.00 |
| spec[5] | 0.99 | 1.00 | 1.00 | 0.01 |
| beta[1,1] | 2.56 | 9.65 | 17.81 | 4.26 |
| beta[2,1] | 2.49 | 9.38 | 16.25 | 3.84 |
| beta[3,1] | 1.69 | 6.28 | 10.96 | 2.64 |
| beta[4,1] | 0.88 | 3.57 | 6.51 | 1.58 |
| beta[5,1] | 0.92 | 3.56 | 6.64 | 1.58 |
| beta[1,2] | -3.49 | -1.80 | -0.70 | 0.86 |
| beta[2,2] | -0.27 | 0.20 | 0.86 | 0.28 |
| beta[3,2] | -0.92 | -0.27 | 0.19 | 0.28 |
| beta[4,2] | -17.39 | -8.57 | -2.86 | 4.09 |
| beta[5,2] | -18.79 | -10.32 | -2.24 | 4.53 |
| sigma[1] | 1.13 | 4.32 | 7.91 | 1.90 |
| sigma[2] | 0.18 | 1.63 | 3.63 | 1.13 |

Finite Mixture Model (class 1 = diseased, class 2 = healthy)

|  | Lower95 | Mean | Upper95 | SD |
| --- | --- | --- | --- | --- |
| prevalence | 0.44 | 0.52 | 0.61 | 0.04 |
| sens[1] | 0.94 | 0.98 | 1.00 | 0.02 |
| sens[2] | 0.95 | 0.98 | 1.00 | 0.01 |
| sens[3] | 0.81 | 0.88 | 0.94 | 0.04 |
| sens[4] | 0.61 | 0.72 | 0.84 | 0.06 |
| sens[5] | 0.61 | 0.72 | 0.83 | 0.06 |
| spec[1] | 0.77 | 0.88 | 0.99 | 0.06 |
| spec[2] | 0.40 | 0.51 | 0.62 | 0.05 |
| spec[3] | 0.46 | 0.56 | 0.65 | 0.05 |
| spec[4] | 0.97 | 0.99 | 1.00 | 0.01 |
| spec[5] | 0.96 | 0.99 | 1.00 | 0.01 |
| w[1,1] | 0.71 | 0.85 | 0.99 | 0.08 |
| w[2,1] | 0.24 | 0.37 | 0.51 | 0.07 |
| w[3,1] | 0.31 | 0.43 | 0.55 | 0.06 |
| w[4,1] | 0.96 | 0.99 | 1.00 | 0.01 |
| w[5,1] | 0.95 | 0.98 | 1.00 | 0.02 |
| w[1,2] | 0.89 | 0.95 | 1.00 | 0.03 |
| w[2,2] | 0.90 | 0.95 | 1.00 | 0.03 |
| w[3,2] | 0.65 | 0.77 | 0.90 | 0.07 |
| w[4,2] | 0.28 | 0.49 | 0.71 | 0.11 |
| w[5,2] | 0.29 | 0.48 | 0.71 | 0.11 |
| eta[1] | 0.10 | 0.22 | 0.33 | 0.06 |
| eta[2] | 0.31 | 0.46 | 0.58 | 0.07 |
